# Role of Insulin-Transferrin-Selenium in Auricular Chondrocyte Proliferation and Engineered Cartilage Formation *in Vitro*

**DOI:** 10.3390/ijms15011525

**Published:** 2014-01-21

**Authors:** Xia Liu, Jinchun Liu, Ning Kang, Li Yan, Qian Wang, Xin Fu, Yuanyuan Zhang, Ran Xiao, Yilin Cao

**Affiliations:** 1 Research Center of Plastic Surgery Hospital, Chinese Academy of Medical Sciences & Peking Union Medical College, Beijing 100144, China; E-Mails: xliu0810@gmail.com (X.L.); B2008183@gmail.com (J.L.); eilain1983@gmail.com (N.K.); yanli102894@gmail.com (L.Y.); liousy6529@gmail.com (Q.W.); xiaoxin8349@gmail.com (X.F.); 2 Wake Forest Institute of Regenerative Medicine, Wake Forest School of Medicine, Winston-Salem, NC 27157, USA; E-Mail: yzhang@wakehealth.edu

**Keywords:** insulin-transferrin-selenium, auricular chondrocyte, dedifferentiation, hypertrophy, serum, engineered cartilage

## Abstract

The goal of this study is to determine the effects of Insulin-Transferrin-Selenium (ITS) on proliferation of auricular chondrocytes and formation of engineered cartilage *in vitro*. Pig auricular monolayer chondrocytes and chondrocyte pellets were cultured in media containing 1% ITS at different concentrations of fetal bovine serum (FBS, 10%, 6%, 2%, 0%), or 10% FBS alone as a control for four weeks. Parameters including cell proliferation in monolayer, wet weight, collagen type I/II/X (Col I, II, X) and glycosaminoglycan (GAG) expression, GAG content of pellets and gene expression associated with cartilage formation/dedifferentiation (lost cartilage phenotype)/hypertrophy within the chondrocyte pellets were assessed. The results showed that chondrocytes proliferation rates increased when FBS concentrations increased (2%, 6%, 10% FBS) in ITS supplemented groups. In addition, 1% ITS plus 10% FBS significantly promoted cell proliferation than 10% FBS alone. No chondrocytes grew in ITS alone medium. 1% ITS plus 10% FBS enhanced cartilage formation in terms of size, wet weight, cartilage specific matrices, and homogeneity, compared to 10% FBS alone group. Furthermore, ITS prevented engineered cartilage from dedifferentiation (*i.e.*, higher index of Col II/Col I mRNA expression and expression of *aggrecan*) and hypertrophy (*i.e.*, lower mRNA expression of *Col X* and *MMP13*). In conclusion, our results indicated that ITS efficiently enhanced auricular chondrocytes proliferation, retained chondrogenic phenotypes, and promoted engineered cartilage formation when combined with FBS, which is potentially used as key supplementation in auricular chondrocytes and engineered cartilage culture.

## 1. Introduction

Auricular cartilage reconstruction is a common plastic surgical approach for congenital auricle deformity or traumatic cartilage damage. Cell-based tissue engineering offers promise in auricular construction [1]. Auricular chondrocyte appears to be the logical choice among the various cell types for auricular cartilage tissue engineering, as chondrocyte is the only single cell type that resides within cartilage and is solely responsible for the synthesis and turnover of the extracellular matrix (ECM). In addition, not all chondrocytes are suitable for auricular cartilage tissue engineering as chondrocytes obtained from other cartilage tissues (such as articular cartilage) may elicit distinct responses during their respective development of a tissue-engineered neocartilage [2]. However, there are challenges to harvest and generate enough amounts of cells for auricular tissue repair. Only small amount of cells can be obtained from the limited size of auricular cartilage. In addition, it is very difficult for the chondrocytes to proliferate and maintain its phenotype due to its dedifferentiation in current culture media. Therefore, to develop an optimal culture media that can promote cells proliferation and maintain their phenotypes in 2D or 3D culture would be desirable for potential in cartilage repair and regeneration.

Basal chondrogenic culture medium is often supplemented with fetal bovine serum (FBS) to enhance cellular activities, such as extracellular matrix synthesis or cell proliferation during chondrocytes cultured in 2D or 3D conditions [3]. However, the chondrocytes frequently dedifferentiate with increasing passages in this traditional culture media with serum [4]. A decrease in elastin expression and morphological changes of hypertrophy were noted in auricular chondrocytes after expansion in monolayer culture [5]. In addition, dedifferentiated chondrocytes produced fewer glycosaminoglycan (GAG) and collagen type II (Col II) [6,7], which resulted in the poor cartilage tissue formation. Furthermore, undesired hypertrophy and mineralization within the engineered tissue change the features of elastic cartilage tissue [8]. These results suggested that basal chondrogenic culture medium with serum is lacks the key factors to prevent these shortages.

There are three types of cartilage tissues, *i.e.*, elastic cartilage, hyaline cartilage, and fibrocartilage. Although each type of chondrocyte requires different supplements in the culture, it might share certain key factors. Insulin-Transferrin-Selenium (ITS), as a compound of insulin, transferring, and sodium selenium, has emerged as a commercially available medium supplement [3]. ITS is often utilized in monolayer culture of chondrocyte from articular cartilage [9–11] and its cartilage tissue engineering [3,9,12,13] as a serum substitute. ITS can prevent articular chondrocyte dedifferentiation in monolayer culture and promote high quality engineered cartilage formation when combined with other growth factors, such as transforming growth factor beta 2 (TGF-β2) [9]. ITS can regulate proliferation [9], and matrix synthesis [3,14,15] of hyaline chondrocytes, but little is known about its action on auricular chondrocytes growth and elastic cartilage formation *in vitro*.

To gain a better understanding of the effects of ITS on the proliferation of auricular chondrocytes and formation of engineered cartilage *in vitro*, we assessed cell proliferation, gross appearance, wet weight, histological changes, GAG content and gene expression associated with cartilage formation, dedifferentiation, and its hypertrophy after porcine auricular chondrocytes and chondrocyte pellets were cultured with ITS supplementation.

## 2. Results and Discussion

### 2.1. Expansion of Auricular Chondrocytes in Monolayer Culture

**Figure 1.**
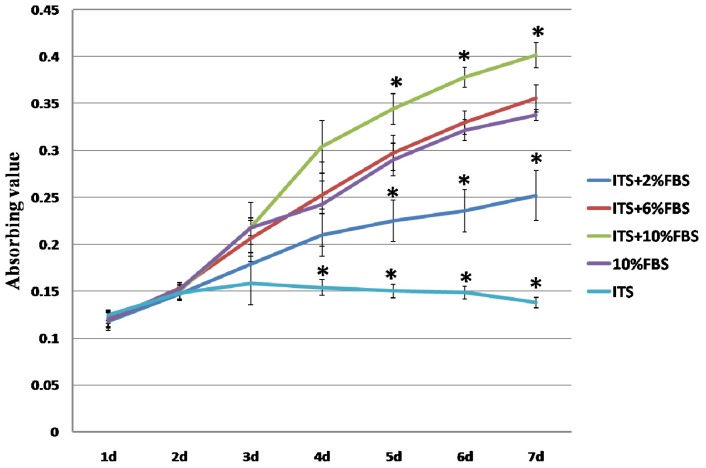
Monolayer growth rates of chondrocytes cultured in different media were determined by MTT assay for seven days. Error bars indicate standard deviations of the mean for three repeats; * Indicate the significant differences compared with all other groups at the same time point; d: day.

Cell proliferation of the chondrocytes cultured in different media was assessed with 3-(4,5)-dimethylthiahiazo(−z-y1)-3,5-di-phenytetrazoliumromide (MTT) (Figure 1). Numbers of cultured cells in 1% ITS + 10% FBS group were significantly higher than in other groups from day five. This result was consistent with that of Chua’s experiment [9], in which 1% ITS + 2% FBS doubled cell numbers of human hyaline chondrocyte compared to medium added with 2% FBS alone. In addition, the cell proliferation in 1% ITS + 6% FBS group was similar to that in 10% FBS alone group. In agreement with the other studies [9,16], the cell growth in 1% ITS + 2% FBS group is lower than that in 10% FBS alone group, and the chondrocytes were unable to grow in serum free medium supplemented with ITS. These results suggested that ITS could enhance auricular chondrocytes growth when combined with FBS, but it alone could not promote chondrocytes proliferate.

### 2.2. Gross View and Wet Weight of Auricular Chondrocyte Pellets

**Figure 2.**
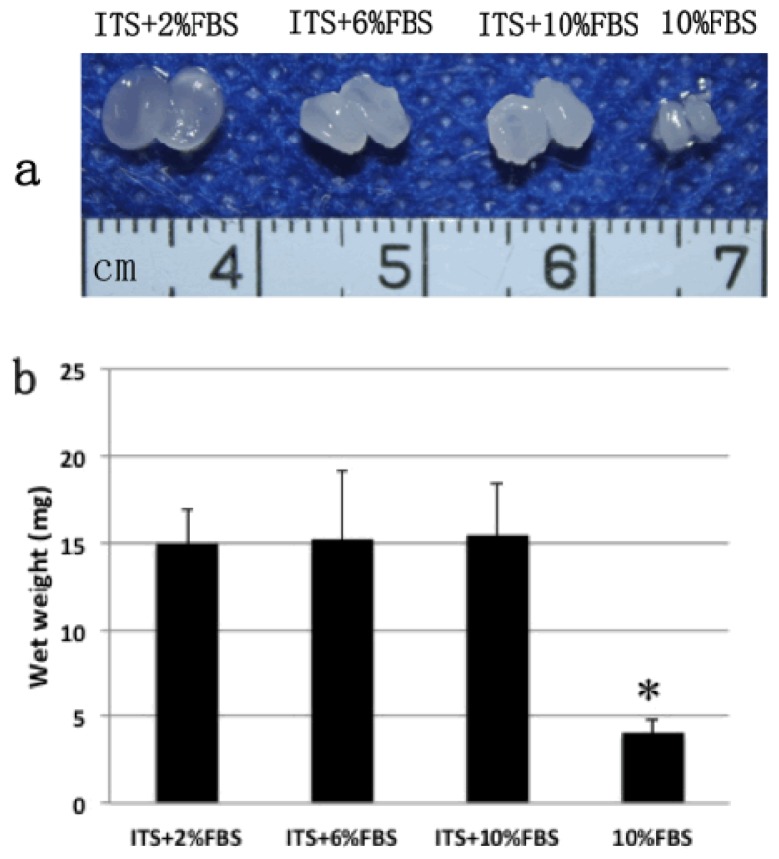
Macroscopic morphology (**a**) and wet weights (**b**) of the pellets cultured in different medium for four weeks. Error bars indicate standard deviation of the mean for three repeats of each group; * Indicate the significant difference compared with all other groups.

After four weeks culture, the chondrocyte pellets formed white, translucent or opaque, cartilage-like tissue in each of the three ITS plus FBS groups or 10% FBS alone group (Figure 2a), but not in ITS alone group, in which the membrane like substance was formed which was hard to be collected. Therefore, there were no wet weight, histology and molecular examination results of ITS alone group. This result was consistent with a previous study in which serum-free ITS-supplemented medium was less effective than ITS-serum-containing medium in stimulating ECM synthesis in chondrocyte-seeded peptide hydrogels [3]. In the current study, we used the scaffold-free culture system to produce the cell pellet that was more close to native cartilage tissue *in vivo*. The pellet can promptly respond to exogenous factors and exclude the effects of scaffold [17].

The pellet sizes in the three ITS plus FBS groups were all similar and significantly larger than that in 10% FBS alone group. The wet weights of the three ITS plus FBS groups were significantly higher (*p* < 0.01) than that of FBS alone group (Figure 2b). No significant difference in wet weight was observed among the three ITS plus FBS groups. These results suggested that ITS can promote the formation of engineered cartilage in the presence of FBS.

### 2.3. Histological and Immunohistochemical Analysis

**Figure 3.**
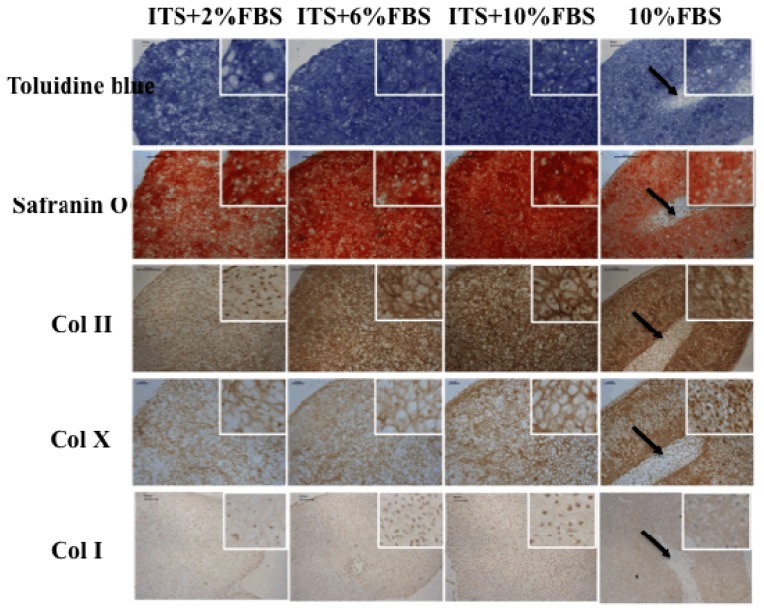
Chondrocyte pellet formation in four groups after four weeks culture, assessed by toluidine blue staining, safranin O staining, immunohisto-chemical staining of Col I/II/X. Black arrows show heterogeneous structures.

Toluidine blue and safranin O staining confirmed GAG expression in the cell pellets. The GAG density is higher in three ITS plus FBS groups than that in FBS alone group (Figure 3, toluidine blue and safranin O). With FBS concentration increasing, the GAG expression appeared stronger. Col II expression displayed the similar fashion to GAG (Figure 3, Col II). The Col II density was higher in chondrocyte pellets cultured in 1% ITS + 10% FBS group than in other groups. For Col I expression, the difference among the groups was not as significant as that of Col II. These results indicated that ITS can promote the production of GAG and Col II, especially when combined with 10% FBS.

Our results also showed that ITS decreased hypertrophy differentiation of auricular chondrocytes when added into the culture medium. Col X is commonly used as one of the markers of hypertrophy differentiation of chondrocytes. Col X expression in the pellets cultured in ITS supplemented groups is lighter than that in FBS alone group (Figure 3, Col X).

In the current study, the chondrocyte pellets were homogenous in three groups with ITS plus FBS, while the pellets were heterogeneous (black arrows in Figure 3) with some fibrous tissues at the center of the pellet in FBS alone group. This indicated that ITS effectively enhanced the thickness and homogeneity of cartilage formation (Figures 2 and 3). The cartilage pellet in the outer part was much easier and quicker to form than that in the inner part because the nutrition distribution and metabolites was better in the outer part of the pellet [18,19]. In addition, the outer compact cartilage matrices may block the transportation of nutrition and byproduct of the inner part during the cartilage formation processes, which eventually lead to insufficiency of cartilage formation with “hollow” formation at the central region [18,20]. The mechanism of ITS in enhancing the thickness and homogeneity of cartilage formation is not clear but it may be involved in progressive effect of ITS on cell proliferation, migration, and nutrition transportation [20]. The matrices in the outer region of the pellets formed in ITS supplemented groups was not as dense as that in FBS alone group, which enhanced the efficient transportation of nutrition into the inner part and thus contributed to homogenous cartilage formation. The accurate mechanism needs to be further verified.

### 2.4. GAG Content

**Figure 4.**
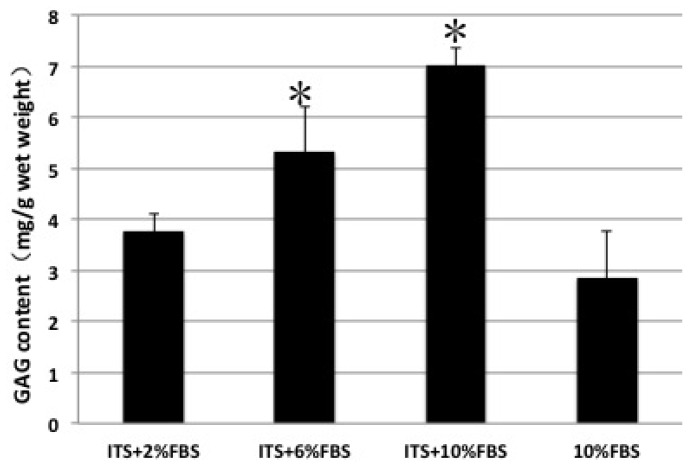
GAG content of the pellets cultured in different medium for four weeks. Error bars indicate standard deviation of the mean for three repeats of each group; * Indicate the significant differences compared with other groups.

The GAG content analysis confirmed the histological results of toluidine blue and safranin O staining. ITS plus 10% FBS group had the highest GAG content and showed a significant difference compared with other groups. Consistent with weak positive staining of toluidine blue and safranin O, 10% FBS alone group showed significantly lower GAG content (Figure 4). These results suggested that ITS enhanced the formation of cartilage specific extracellular matrix.

### 2.5. Quantitative Gene Expression of Chondrocyte Pellets

**Figure 5.**
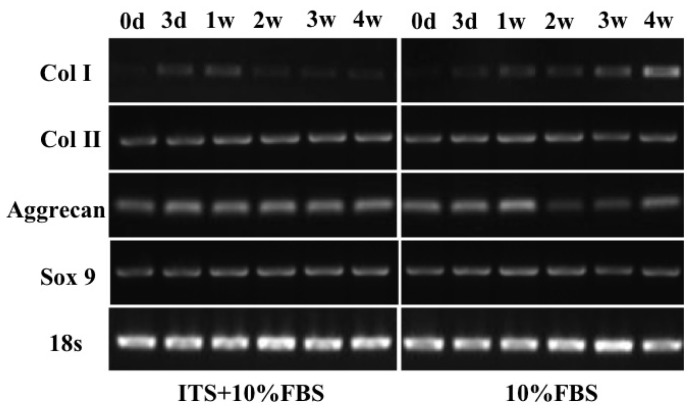
Gene expression of *Col I*, *Col II*, *Aggrecan*, and *Sox 9* in chondrocyte pellets cultured for four weeks in two groups, assessed by semi-quantitative PCR. d: day; w: week.

To further verify the effects of ITS on cartilage formation, the gene expression related to cartilage formation, dedifferentiation and hypertrophy was assessed in each group at different time points (0 day, 3 day, 1 week, 2 weeks, 3 weeks, 4 weeks). Semi-quantitative PCR analysis (Figure 5) showed that the mRNA expression levels of *Col II*, *aggrecan* and *Sox 9* in ITS + 10% FBS group were higher with increasing time. While in the 10% FBS alone group, there was a higher expression of *Col II*, *aggrecan* and *Sox 9* in the early time (one week). The early formation of large quantity of ECM may affect the nutrition transportation, which may explain the heterogeneous structure formation in the central area of the pellet in 10% FBS alone group.

**Figure 6.**
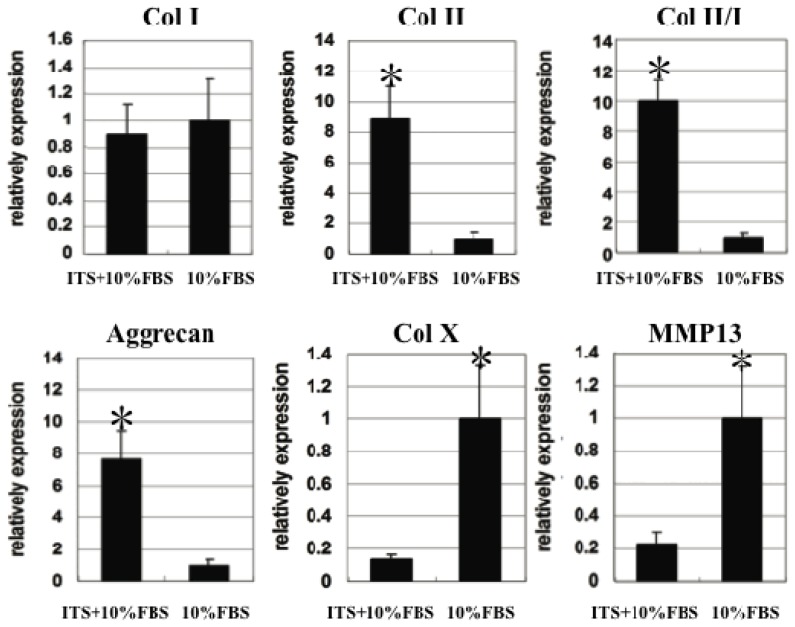
Quantitative real-time PCR results of *Col I*, *Col II*, *Col X*, *aggrecan*, and *MMP13* expression in chondrocyte pellets cultured for four weeks in two groups. * Indicate significant differences.

In order to show the gene expression differences quantitatively, we did quantitative real-time PCR at the fourth week. The results (Figure 6) showed the significant difference between the two groups in the expression of *Col II* and *aggrecan* that was agreed with the results of Figure 5, while the difference of *Col I* expression was not as apparent, as shown in Figure 5. Traditional PCR methods use agarose gels for detection of PCR amplification at the final phase or end-point of the PCR reaction. The end point is variable from sample to sample. In contrast, the results of real-time PCR are more precise. ITS supplementation resulted in significant higher Col II/Col I index in tissue engineered cartilage formation, indicating that ITS was necessary to reduce the chondrocyte dedifferentiation process.

Chondrocyte pellets in this study also showed tendency of being hypertrophic since Col X and MMP13 were detected. Terminal differentiation of chondrocytes into hypertrophic cells is an obligatory step in the endochondral ossification pathway. Chondrocytes in adult auricular cartilage rarely express Col X. However, *Col X* expression could occur and inevitably trigger the terminal differentiation after a long time culture of chondrocytes, even in 3D culture [8]. In this study, supplementation of 1% ITS in 10% FBS containing medium could reduce expression of hypertrophy-related genes, such as *Col X* and *MMP13*, indicating an inhibitory effect of ITS on hypertrophic differentiation.

All these results confirmed that ITS supplementation resulted in higher quality of engineered cartilage formation by scoring higher cartilage differentiation index and lower dedifferentiation and hypertrophy index.

ITS has been used as a serum substitute in low serum or serum free media in chondrocyte culture [14,21,22]. However, it should be noted that serum consists of rich variety of growth factors that is benefit for cell proliferation and tissue formation. High concentration serum (10% FBS) can induce cell division and extracellular matrix synthesis to support the development of good quality tissue substitutes. In our study, ITS alone did neither induce cell proliferate nor promote the chondrocyte pellet formation, however, when combined with FBS, with the increase of FBS concentration cell proliferation and quality of engineered cartilage increased. Our results verified that both ITS and serum are key factors in auricular chondrocytes culture and cartilage formation.

## 3. Experimental Section

### 3.1. Auricular Chondrocytes Isolation and Monolayer Culture

Auricular cartilage tissue (~2 × 3 cm^2^) was obtained from the external ears of 3 healthy mini (male) pigs (aged 3 months, weight 10–15 kg). Institutional Animal Care and Use Committee of Plastic Surgery Hospital approved animal experiments.

Cartilage specimens were minced into ~1 mm^3^ pieces, washed in phosphate buffer saline (PBS; pH 7.2; Gibco, Grand Island, NY, USA), and digested with 0.25% (*w*/*v*) trypsin (Gibco, Grand Island, NY, USA) for half an hour followed by 0.15% (*w*/*v*) collagenase IV (Sigma-Aldrich, St. Louis, MO, USA) overnight at 37 °C in a shaker. After filtration through a nylon mesh (200 μm), the cells were washed, counted, and seeded at a density of 2500 cells/cm^2^ into a 10 cm cell culture dish (Corning, Corning, NY, USA) in DMEM medium (Gibco, Grand Island, NY, USA) containing 10% FBS, 100 U/mL penicillin (Gibco, Grand Island, NY, USA), and 100 μg/mL streptomycin (Gibco, Grand Island, NY, USA). All cultures were maintained in a 5% CO_2_ incubator (Thermo, Waltham, MA, USA) at 37 °C with culture medium changed every 3 days. Chondrocytes at primary culture were grown until confluent and then sub-cultured using 0.05% trypsin-EDTA. Chondrocytes of passage 2 were harvested for further use.

### 3.2. Chondrocyte Pellets Culture

Aliquots of 5 × 10^5^ cells were centrifuged at 931 g for 8 min in 15 mL polypropylene conical tubes [23]. After 24 h of incubation, the sediment cells formed a spherical aggregate at the bottom of each tube. Pellets were divided into five groups and cultured in different media, as listed in Table 1.

### 3.3. Proliferation of Chondrocytes

The chondrocytes at passage 2 were cultured in five different culture media as described in Table 1. Proliferation of chondrocytes was estimated by MTT assay [24].

### 3.4. Histology and Immunohistochemistry

After culture for 4 weeks, pellets in all groups (3 pellets/group) were harvested and fixed in 4% paraformaldehyde overnight at 4 °C, embedded in paraffin, and sectioned into 5 mm sections. The sections were stained with safranin O and toluidine blue staining to evaluate the histological structure and proteoglycans.

For immunohistochemistry, the slides were processed the standard program of dewaxing, rehydrating, block endogenous peroxidase 10 min in 3% H_2_O_2_ (Zhongshan Golden Bridge Biotechnology, Beijing, China), followed by a digestion with pepsin (ZLI-9013 Zhongshan Golden Bridge Biotechnology, Beijing, China) for antigen retrieval. Nonspecific signals were blocked by incubation with 1% normal goat serum (1% BSA 1:10 dilution) for 10 min. The sections were then incubated with rabbit polyclonal antibody to Col I (1:200, Abcam, Cambridge, MA, USA), mouse monoclonal antibody to Col II (1:200, Sigma, St. Louis, MO, USA) or rabbit antibody to Col X (1:200, Abcam, Cambridge, MA, USA), respectively. The antigen-antibody complex was visualized with horseradish peroxidase (HRP)-conjugated anti-mouse/rabbit antibody followed by color development with diaminobenzidene (DAB) substrate kit (DAB-kit, Zhongshan Golden Bridge Biotechnology, Beijing, China).

### 3.5. GAG Quantification

The proteoglycan content was evaluated by GAG quantification using spectrophotometric micro determination with dimethylmethylene blue dye, as previously described [25,26]. Briefly, GAG was precipitated by guanidinium chloride (Gncl) solution. After dissolving the GAG precipitate, the optical density (OD) was determined at 595 nm. A standard curve was established using chondroitin-4-sulfate, and the total GAG amounts were determined from the OD value, which correlated to the corresponding GAG amount in the standard curve.

### 3.6. RNA Extraction and Semi-Quantitative and Quantitative Real-Time Polymerase Chain Reaction (PCR)

Pellets in ITS + 10% FBS group and 10% FBS alone group were collected at different time points (0 day, 3 days, 1 week, 2 weeks, 3 weeks, 4 weeks; 3 pellets/group/time point) and then minced. Total RNA was extracted using TRIzol reagent (Invitrogen, Carlsbad, CA, USA) according to the manufacturer’s instructions. RNA was reverse transcribed into cDNA.

For semi-quantitative PCR analyses, the cycling reactions were processed in a peltier thermal cycler (DNA Engine^®^, Bio-RAD, Hercules, CA, USA) and the RT-PCR products were visualized using Gel Image System (Biospectrun AC, UVP, Upland, CA, USA).

For quantitative real-time PCR, the expression levels of selected genes were analyzed using a LightCycler 480 system with a SYBR green kit (Roche Molecular Biochemical, Mannheim, Germany).

The primer sequences used in this study are shown in Table 2. Primer sequences were quoted from references [27,28] or designed using Gene Runner software (version 3.05, Hastings Software Inc. Hastings, NY, USA). The specificity of sequences was verified using the basic local alignment search tool (BLAST) of the National Center for Biotechnology Information (NCBI) online database. The expression level of each gene was normalized to *GAPDH*.

### 3.7. Statistical Analysis

All numerical data were presented as mean ± standard error of the mean. The difference between groups was evaluated by one-way analysis of variance (ANOVA). In addition, comparisons among the various groups were performed using *post hoc* pair-wise comparisons. Analysis was performed with SPSS software (version SPSS 11.0, SPSS Inc, Chicago, IL, USA) and *p* < 0.05 was considered to be significant.

## 4. Conclusions

Our study suggested that ITS enhanced the auricular chondrocyte proliferation; promoted production of cartilage-specific matrix and homogeneity in the presence of serum. Furthermore, ITS inhibited the gene and protein expression related with dedifferentiation and hypertrophic differentiation of the chondrocytes. Therefore, ITS appears a key factor with 10% serum for the auricular chondrocyte proliferation and high quality engineered cartilage formation *in vitro*.

## Figures and Tables

**Table 1.** Auricular chondrocytes cultured in different media.

Groups	Abbreviation of Groups	Culture Media
1	ITS + 2% FBS	DMEM + 1% ITS + 2% FBS
2	ITS + 6% FBS	DMEM + 1% ITS + 6% FBS
3	ITS + 10% FBS	DMEM + 1% ITS + 10% FBS
4	10% FBS	DMEM +10% FBS
5	ITS	DMEM + 1% ITS

DMEM, Dulbecco’s Modified Eagle Medium (Gibco); FBS, fetal bovine serum (Gibco); ITS, 10 μg/mL insulin + 5.5 μg/mL transferrin + 6.7 ng/mL sodium selenite (M&C Technology).

**Table 2.** Primer sequences used in semi-quantitative and quantitative real-time polymerase chain reaction (PCR) for gene expression analysis.

Gene	Primer 5′–3′	Size (bp)
*Col I* [27]	F: CGATGGCTGCACGAGTCACAC R: CAGGTTGGGATGGAGGGAGTTTAC	180
*Col II* [27]	F: CCGGGCAGAGGGCAATAGCAGGTT R: CAATGATGGGGAGGCGTGAG	128
*aggrecan* [27]	F: CCAGAATCTAGCAGGGAGTCATC R: AGGCAGAGGTGGCTTCAGTC	117
*Sox 9*	F: CACAGCTCACCAGACCTTGA R: GTGGGTTCGAGTTGCCTTTA	176
*Col X* [28]	F: GCTGCCACATTCTGACACAATC R: TGCGCTGAGCATCATTTGAGAC	225
*MMP13*	F: CTTGTTTCTTGTTGCTGCCC R: GTTGGGGTCTTCATCTCCTG	178
*GAPDH* [27]	F: CTGCCCCTTCTGCTGATGC R: TCCACGATGCCGAAGTTGTC	151
*18S*	F: TGAGAAACGGCTACCACATC R: TCCCAAGATCCAACTACGAG	250
